# Cyanotoxin Analysis and Amino Acid Profiles of Cyanobacterial Food Items from Chad

**DOI:** 10.1007/s12640-020-00240-x

**Published:** 2020-07-11

**Authors:** J. S. Metcalf, R. A. Dunlop, S. A. Banack, N. R. Souza, P. A. Cox

**Affiliations:** Brain Chemistry Labs, Box 3464, Jackson, WY 83001 USA

**Keywords:** Chad, Cyanobacteria, Toxins, Nutrition, Microcystin, Neurodegenerative disease

## Abstract

In some parts of the world, cyanobacteria are used as a food in the human diet, due to their ready availability. Lake Chad, has long been a traditional site for the collection of *Arthrospira fusiformis* which is dried and processed at the lake into thin wafers called *Dihé* for later consumption or is transported to market for sale. However, *Dihé* purchased from markets in Chad has not been analyzed for known cyanobacterial toxins or assessed for total amino acid content. Since BMAA in traditional foodstuffs of the indigenous Chamorro people of Guam causes neurodegenerative illness, it is important that *Dihé* from Chad be analyzed for this neurotoxin. BMAA and its isomer AEG were not detected in our analyses, but a further isomer DAB was detected as both a free and bound amino acid, with an increase in the free concentration after acid hydrolysis of this fraction. Microcystins were present in 6 samples at up to 20 μg/g according to UPLC-PDA, although their presence could not be confirmed using PCR for known microcystin synthetic genes. Amino acid analysis of the cyanobacterial material from Chad showed the presence of large amounts of canonical amino acids, suggesting that this may supplement indigenous people on low protein diets, although regular monitoring of the foodstuffs for the presence of cyanotoxins should be performed.

## Introduction

Cyanobacteria, photosynthetic Gram-negative bacteria which appeared early in the earth’s geological record, are essential for ecosystem function and health (Stal 2007). However, when environmental conditions allow, such as with the provision of excess nutrients, then mass populations of cyanobacteria can occur (Smith et al. 1999). Often termed cyanobacterial blooms and scums, they can be unsightly and may produce taste and odor compounds, in addition to being capable of producing a wide range of highly toxic compounds that have been linked to both long- and short-term adverse human health events (Metcalf and Codd 2012; Wood 2016; Mello et al. 2018; Janssen 2019). Many cyanobacterial genera are capable of producing toxins, largely delineated according to their mode of action. These include the microcystins and nodularins, cyclic peptide hepatotoxins, cylindrospermopsins, cytotoxic guanidine alkaloids and the alkaloid neurotoxins, anatoxin-a and anatoxin-a(S), as examples (Metcalf and Codd 2012; Wood 2016; Mello et al. 2018; Huisman et al. 2018). In marine and freshwater environments where cyanobacteria are common, such toxins can be highly toxic to aquatic organisms (Sun et al. 2012; Paerl et al. 2016; Huisman et al. 2018; Gene et al. 2019; Shahmohamadloo et al. 2020).

Although cyanobacteria have the potential to produce toxins, increasingly genera such as *Aphanizomenon* and *Spirulina* (potentially including *Arthrospira*) are being promoted as health food supplements (Bishop and Zubeck 2012). Toxin analysis of such supplements has indicated that they are capable of producing a range of toxins, including microcystins, anatoxin-a and β-*N*-methylamino-L-alanine (BMAA) (Rawn et al. 2007; Rellán et al. 2009; Heussner et al. 2012; Glover et al. 2015; Roy-LaChapelle et al. 2017). The presence of toxins in health food supplements is a potential risk to consumers, such that in the State of Oregon, commercial products of *Aphanizomenon flos-aquae* are legislated to not contain microcystins at a concentration of greater than 1 μg/g (Marsan et al. 2017).

**Fig. 1:**
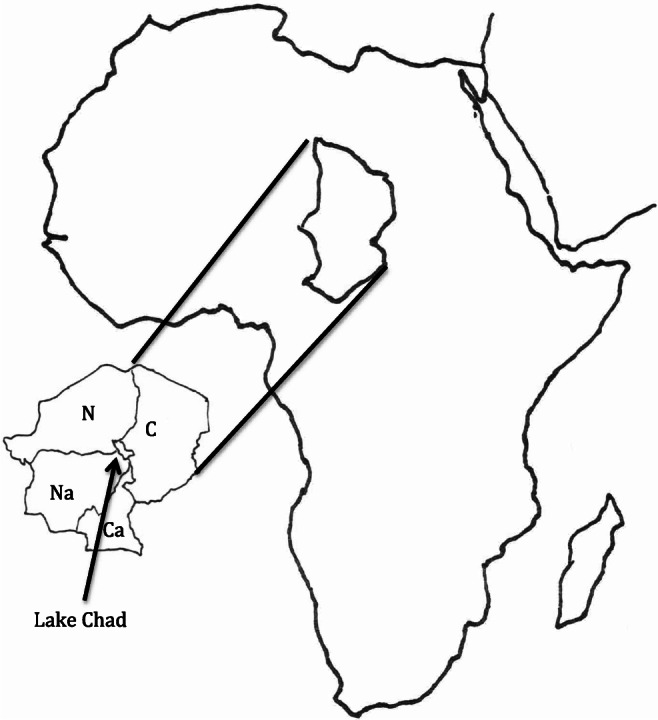
Location of Lake Chad. C, Chad; N, Niger; Na, Nigeria; Ca, Cameroon

**Fig. 2:**
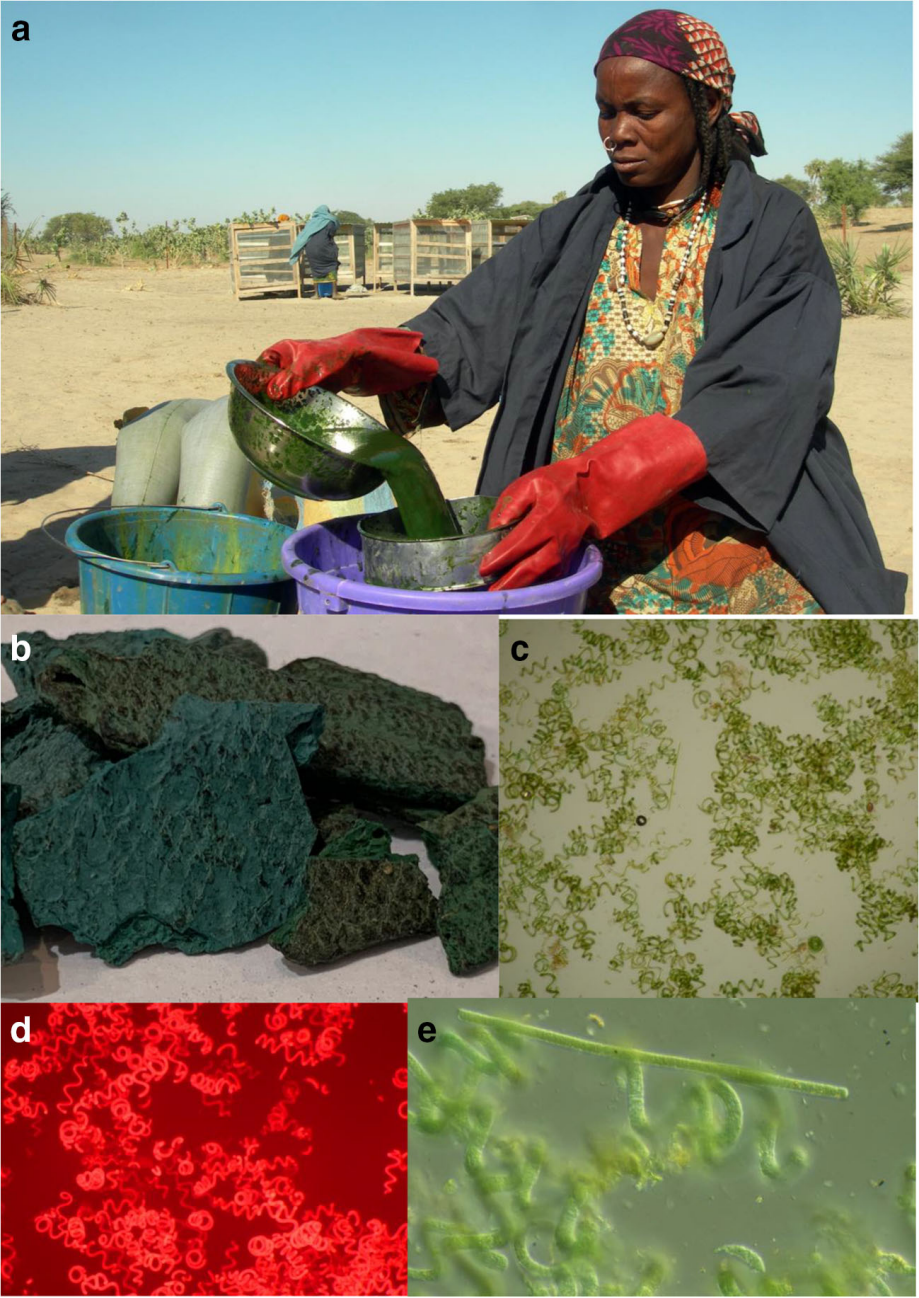
Processed cyanobacterial material from Chad. **a** Arsa Abdoulaye nicknamed “Banaa” pours into a bucket, *Arthrospira*, a dietary supplement (both for human and animal nutrition), in Brandji, on Lake Chad on December 12, 2009. (Photo credit Patrick Fort/AFP via Getty Images). **b** Processed cyanobacterial mats. **c** Light microscopy of *Arthrospira* material. **d** Fluorescence microscopy of *Arthrospira* material. **e** Straight and coiled filaments of *Arthrospira*

The Republic of Chad is a landlocked African country, and the population of this country is considered to be undernourished and in poverty (World Food Programme 2019). Within this country, Lake Chad (Fig. 1) naturally supports blooms of a cyanobacterium identified by Abulqader et al. (2000) as *Arthrospira platensis*, now named *Arthrospira fusiformis* (Voronichin; Komárek et Lund 1990; Sili et al. 2012). This photosynthetic, non-nitrogen fixing prokaryote is harvested by local indigenous women and bloom material is processed for subsequent consumption and sale (Fig. 2a), an activity that is believed to date back to perhaps the ninth century (Abulqader et al. 2000). In Lake Chad, cyanobacteria are harvested from the lake, sun dried, and prepared as thin green wafers called *Dihé* for direct consumption or to be powdered as an ingredient for a sauce, some of which are flavored, to be served on balls of cooked millet or maize (Dangeard 1940, Delpeuch et al. 1975; Ciferri 1983; GCP/CHD/029/EC 2016). Léonard and Compère reported that consumption is mostly restricted to populations northeast of Lake Chad composed primarily of the Kanebou people (where the original sample described by Dangeard originated), but that it is not eaten by fishermen and nomadic people immediately adjacent to Lake Chad or by indigenous peoples near other lakes with abundant *Arthrospira fusiformis* populations (Léonard and Compère 1967, Ciferri 1983). This finding that *Arthrospira fusiformis* is consumed by only a minority of the population of Chad was reconfirmed by a careful survey by Delpeuch et al. (1975) who wrote:

“Consumption is limited to a small part of the Kanem population.

… the frequencies of use vary between one and six meals out of ten; the quantities consumed per person during meals, in the sauce accompanying the millet, are between 9 and 13 g.” (Delpeuch et al. 1975, p. 497).

*Arthrospira* is processed on the shores of Lake Chad for sale in the market (Abulqader et al. 2000) from where we obtained our samples. The wind-blown cyanobacterial scum along the lake shore is gathered and filtered into clay vessels, and then transported to nearby dunes, where the cyanobacterial material is poured into small depressions in the sand for sun drying (Delpeuch et al. 1975). *Arthrospira fusiformis* from Lake Chad is claimed to offer significant nutritional benefits to the indigenous people, including as a source of β-carotene (Carcea et al. 2015, Soudy et al. 2018), and there is some indication that this particular species has antiviral properties (Sharaf et al. 2013). Since *Arthrospira fusiformis* from Lake Chad has been proposed for economic development (Charpy et al. 2008, CP/CHD/029/EC, 2016), a cyanotoxin analysis of market samples is needed.

The presence of *Arthrospira* as a common component of waters in Africa is well known, mostly from the African Rift Valley Lakes Nakuru, Bogoria and Elmenteita in Kenya and of laboratory isolates from Mozambique. A number of cyanobacterial toxins have been identified in cyanobacterial material and/or clinical materials from these sites and include microcystins, anatoxin-a and BMAA as examples (Krienitz et al. 2003; Ballot et al. 2004; Mussagy et al. 2006; Metcalf et al. 2006, 2013).

Concerning Lake Chad, no research has investigated the potential for the presence of toxins in cyanobacterial material and its potential amino acid nutritional quality. A question of particular interest is the possible presence of BMAA in *Arthrospira fusiformis* consumed by indigenous people in Chad (Charpy et al. 2008). BMAA is a non-protein amino acid produced by cyanobacteria (Cox et al. 2005) that contaminates traditional food items in the Pacific island of Guam (Cox et al. 2003; Banack et al. 2006; Banack and Murch 2009) and has been implicated as a cause of a fatal progressive neurodegenerative disease among the indigenous Chamorro people there (Cox et al. 2016; Davis et al. 2020). At its peak, 25% of adults in two villages in Guam perished from amyotrophic lateral sclerosis/parkinsonism dementia complex (ALS/PDC) (Cox and Sacks 2002; Cox et al. 2003). Similar patterns of progressive neurodegenerative disease have not been reported from Chad, although on average the inhabitants of Chad have twice the number of disability-adjusted life-years due to neurological disorders than the inhabitants of Guam (Feigin et al. 2017). Since there has not been a careful survey of ALS/PDC type-diseases in Chad, it is possible that an increased incidence of progressive neurodegenerative disease there has gone unnoticed, but it is also possible that BMAA does not contaminate dried cakes and other traditional food items in Chad made from *Arthrospira*, perhaps due to abundant nitrogen in the lake water which decreases BMAA production (Downing et al. 2011). Alternatively, there may be taxonomic differences in the *Arthrospira* found in Lake Chad compared to other species in this genus (Cox et al. 2005). The purpose of this study was to evaluate the cyanotoxin content of *Arthrospira* in *Dihé* consumed by the indigenous people of Chad and to quantify the amino acids in this food staple. Cyanotoxin analysis was performed using liquid chromatography, mass spectrometry, enzyme inhibition assays, and polymerase chain reaction (PCR) to provide a broader understanding of the potential for toxin production in this cyanobacterial material. Furthermore, cyanobacterial material from Lake Chad is a commonly-consumed local food that may have great nutritional value. Therefore, we wished to determine whether this indigenous food source was potentially beneficial for people who are considered in poverty and undernourished, and whether there was a risk of short and/or long-term intoxication from the presence of toxic compounds.

## Materials and Methods

### Extraction of Cyanobacterial Material

*Dihé* wafers collected and processed from cyanobacteria in Lake Chad were purchased at markets approximately 50–80 km distant from the lake in July and August 2017 (see Table 1 for markets). Upon receipt at the Brain Chemistry Labs (Jackson, WY), a small piece was removed and resuspended with DQ water for visual assessment of cyanobacteria by light and fluorescence microscopy using a Zeiss Axioplan 2 fluorescence microscope (Fig. 2b–e). Cyanobacteria were identified to generic level according to Whitton (2002) and found to be consistent with *Arthrospira* as previously identified by Abulqader et al. (2000). As the samples purchased from the markets were dry, no further processing was performed prior to extraction of cyanotoxins (50 mg/ml), amino acids, and DNA. For microcystins, an aliquot was weighed and extracted with 70% (v/v) methanol with ultrasonication. Samples were left to stand at room temperature for 1 h, before centrifugation and analysis of the supernatant. For cylindrospermopsin and anatoxin-a, subsamples of the material were weighed and extracted with either DQ water or 100% methanol. The material was ultrasonicated and allowed to stand at room temperature for 1 h before centrifugation. The DQ extracts were stored at − 20 °C until analyzed, and the methanol extracts were dried in a SpeedVac and stored at − 20 °C. For BMAA and isomers, aliquots of the cyanobacterial material were weighed and extracted using 20% (w/v) trichloroacetic acid (TCA) with sonication. The samples were left overnight at 4 °C and were centrifuged the following day. A subsample of the TCA extract was added to an equal volume of 12 M HCl for digestion at 110 °C for 16 h. The pelleted material was resuspended with 6 M HCl and underwent digestion at 110 °C for 16 h. The TCA and HCl extracts were centrifuge filtered (0.2 μm) and dried in a SpeedVac and stored at − 20 °C until analysis. For amino acid extraction, aliquots of the material were weighed and hydrolyzed with 6 M HCl for 16 h at 110 °C. Aliquots were centrifuge filtered, dried in a SpeedVac, and stored dry at − 20 °C until analysis.

**Table 1:** Microscopic analysis of cyanobacterial food supplements from Chad (2017)

Sample	Location/date	Microscopy
1	Koundoul #1 08/14	*A,d*
2	NDjaweng souk 08/12	*A*
3	Abena #1 08/07	*A,d*
4	Chagoua #1 07/25	*A,f*
5	Dembe #1 07/15	*A*
6	Moursal market #1 07/09	*A,f*
7	Koundoul #2 08/14	*A,f*
8	Abena #2 08/07	*A,f*
9	Dembe #2 07/15	*A,d,D,f*
10	Chagoua #2 07/25	*A,f*
11	Moursal market #2 07/09	*A,f*
12	NDjaweng souk 08/12	*A,D*

*A* Arthrospira, *d* cell debris, *f* straight cyanobacterial filaments, *D* diatoms

### RT-PCR

Crust samples were weighed into 1.5 mL PCR-clean Eppendorf tubes, the weights recorded, then 500 μL of lysis buffer (100 mM Tris HCl, 5 mM EDTA, 0.2% SDS, 200 mM NaCl, pH 9.0) was added to each tube. Proteinase K was added to each tube at a final concentration of 1 μg/mL (from stock of 100 μg/mL), and samples were incubated at 55 °C for 1 h with gentle shaking. Following incubation, samples were centrifuged (5 min, 10,000×*g*, RT), the supernatant transferred to a new tube and 500 μL phenol/chloroform/isoamyl alcohol added. Tubes were vortexed, then centrifuged (5 min, 10,000×*g*, RT), and the aqueous layer was transferred to a fresh tube, 500 μL chloroform was added, and tubes mixed by inverting several times. Tubes were centrifuged again (5 min, 10,000×*g*, RT) and the supernatant transferred to a fresh tube. Twenty five microliters of 5 M NaCl was added to the supernatant, the tubes mixed by inversion, then 1 mL of 95% ethanol was added, and mixed well. Tubes were incubated at − 20 °C overnight to allow the DNA to precipitate. Following ethanol precipitation, the tubes were centrifuged (5 min, 10,000×*g*, RT), the supernatant was removed and the pellets allowed to air-dry for 20–30 min. Once all the ethanol had evaporated, the pellets were resuspended in nuclease-free water and the DNA concentration measured on a NanoDrop™ 2000 Spectrophotometer (Thermo Fisher Scientific).

Custom primers for *mcy*D (microcystin synthetase) were synthesized by Sigma-Aldrich Custom Oligos for two amplicons, 818 bp; forward (5′–3′), GATCCGATTGAATTAGAAAG and reverse (5′–3′), GTATTCCCCAAGATTGCC (Rantala et al. 2004) and 297 bp; forward 5′-GGTTCGCCTGGTCAAAGTAA-3′ and reverse, 5′-CCTCGCTAAAGAAGGGTTGA-3′ (Kaebernick et al. 2000). RT PCR was conducted using the HotStarTaq® DNA polymerase kit (QIAGEN, Cat No./ID: 203203) using 50 ng DNA and in accordance with the manufacturer’s instructions using the following conditions; initial denaturation for 15 min, 95 °C; then 35 cycles of 30 s, 94 °C; 30 s, (56 °C, 818 bp amplicon, 60 °C, 297 bp amplicon), 1 min, 72 °C, and final extension for 10 min at 72 °C. The PCR product (1 μL) was run on a 2% agarose gel in 1× TAE with 2.5% GelStar™ Nucleic Acid Gel Stain (Lonza Bioscience, Cat No./ID: 50535) for approximately 45 min or until the dye front reached the bottom of the gel. The gel was imaged on a BioRad ChemiDoc in the UV channel. A positive control for *mcy*D, *Microcystis* PCC7813 (Pasteur Culture Collection, Paris, France), was simultaneously extracted, transcribed (10 ng), and run in the gel, as described above. Conditions were optimized using a temperature gradient during the reverse transcription and DNA concentrations from 1 to 500 ng.

### UPLC-PDA

Dried extracts were resuspended in their respective solvent for ultra-performance liquid chromatography with photodiode array (UPLC-PDA) analysis of known cyanotoxins using a Waters Acquity UPLC system (Metcalf et al. 2018). Microcystins (70% v/v methanol), cylindrospermopsin (DQ water), and anatoxin-a (DQ water) were assessed by comparison to known MC-LR, cylindrospermopsin, and anatoxin-a standards with PDA spectral matching at 238 nm, 262 nm, and 227 nm, respectively.

### Triple-Quadrupole Mass Spectrometry

Dried free and protein bound samples were resuspended with DQ water and derivatized with 6-aminoquinolyl-N-hydroxysuccinimidyl carbamate (AQC) for triple-quadrupole mass spectrometry (Metcalf et al. 2018; Banack 2020). Further samples underwent solid-phase extraction (SPE; Metcalf et al. 2013) to concentrate samples and remove contaminating compounds. After elution, the samples were dried in a rotary evaporator and resuspended with 500 μl 20 mM HCl. These were again derivatized with AQC and underwent triple-quadrupole mass spectrometry for BMAA and isomers using the validated AOAC method with slight modifications for optimization in a different laboratory (Glover et al. 2015; Banack 2020). LOD was calculated at 0.01 ng/ml, and LLOQ was calculated to be 0.04 ng/ml for all analytes using FDA guidelines (FDA 2018).

### Acetylcholine Esterase Inhibition Assay

Aqueous extracts of cyanobacterial material underwent preliminary assessment of anatoxin-a(*S*) content using a colorimetric acetylcholine esterase inhibition assay with reference to a neostigmine standard curve (Metcalf et al. 2018).

### Amino Acid Analysis of Cyanobacterial Material

The dried total acid hydrolysate aliquot (100 μl) was resuspended to the original volume with 20 mM HCl and then diluted 1/10 with 20 mM HCl for amino acid analysis using a Hitachi L-9800 amino acid analyzer using a PH buffer set (PH-1/AN0-8706; PH-2/AN0-8707; PH-3/AN0-8708; PH-4/AN0-8709; PH-RG/AN0-8710) and column (L8900PH-8554514) following a method according to Cox and Metcalf (2017). Amino acids were quantified with reference to standards of the 20 canonical amino acids.

## Results

Light microscopy of the Chad cyanobacterial material showed the presence of *Arthrospira* as the principal cyanobacterium to be present (Table 1; Fig. 2b–d). Analysis of the cyanobacterial material by UPLC-PDA did not identify cylindrospermopsin or anatoxin-a, with trace amounts of anatoxin-a(*S*) in four samples according to acetylcholine esterase inhibition assay (Table 2). Analysis by UPLC-PDA indicated the presence of microcystin variants at up to 20 μg/g in six samples, which were not identified further due to their low concentration. Analysis by PCR for microcystin synthetase did not detect genes for *mcy*D. When the extracts were analyzed for BMAA and isomers, no BMAA or AEG were detected. However, DAB was present as both a free and bound compound. Furthermore, when the free TCA extract was hydrolyzed, the amount of DAB detected was found to greatly increase, suggesting that DAB may be present as a bound component of small peptides or other compounds (Table 2).

**Table 2:** Cyanotoxin analysis of cyanobacterial food supplements from Chad (2017). All values expressed as μg/g

Sample	Anatoxin-a(S)	Microcystin	Free DAB	Hydrolysed free DAB	Bound DAB
1	T	ND	0.89	72.05	2.16
2	ND	19.6 (3)	0.17	3.60	1.59
3	ND	ND	1.16	64.15	5.02
4	ND	ND	1.50	18.34	2.54
5	ND	ND	0.20	6.10	1.59
6	T	ND	1.14	15.93	1.80
7	ND	1.48	2.21	28.89	1.32
8	ND	1.59	1.15	21.53	1.08
9	T	1.28	0.81	19.73	1.15
10	ND	0.49	2.24	36.24	1.38
11	T	4.72	1.33	31.64	1.29
12	ND	ND	ND	13.87	0.82

Numbers in parentheses represent no. variants

*T* trace, *ND* not detected

Amino acid analysis showed the presence of the majority of the 20 canonical amino acids in the cyanobacterial material, ranging from not detectable to around 50 μg/mg for glutamate (Table 3). The order of abundance of amino acids was Glu>Tyr>Trp>Asp>Ala>Arg>Val>Thr>Gly>Leu>Ser>Lys>Pro>Phe>His>Cys>Met>Ile>Gln>Asn.

**Table 3:** Amino acid analysis of cyanobacterial food supplements from Chad. Values are expressed as μg/mg dry weight. Figures in parentheses show the recommended daily intake of essential amino acids for adults (mg/kg; WHO, 2007)

	Chad supplement													
Amino acid	1	2	3	4	5	6	7	8	9	10	11	12	Mean	Rank
Asp	26.2	25.7	25.2	19.6	23.6	19.6	22.4	25.0	20.0	23.4	21.7	23.6	23.0	4
Thr	15.9	16.2	15.6	12.1	14.4	12.3	13.1	14.5	12.1	13.8	13.2	14.2	14.0	8
Ser	1.5	15.9	14.3	11.1	13.3	10.7	12.5	14.1	10.4	13.2	11.6	13.5	11.8	11
Asn	ND	ND	ND	ND	ND	ND	ND	ND	ND	ND	ND	ND	ND	20
Glu	49.1	51.6	46.7	41.0	19.2	40.4	47.3	53.3	39.8	49.0	43.8	46.0	43.9	1
Gln	ND	0.2	0.3	ND	0.3	0.3	0.4	0.5	0.4	0.5	0.5	0.3	0.4	19
Cys (4.1)^*^	2.6	0.4	5.5	2.8	5.0	5.0	3.1	2.7	9.0	3.3	9.0	1.5	4.2	16
Pro	5.8	1.4	6.4	12.6	6.7	16.3	8.0	6.0	21.3	8.7	18.4	5.0	9.7	13
Gly	14.8	15.1	14.6	11.0	13.2	11.0	12.2	13.5	11.0	12.7	12.0	13.3	12.9	9
Ala	25.5	23.6	23.8	17.9	21.8	17.2	19.2	21.3	17.3	20.4	19.1	21.1	20.7	5
Val	17.9	18.4	17.6	13.5	16.2	13.7	14.9	16.9	14.0	16.1	15.3	16.3	15.9	7
Met (10.4)^*^	2.9	4.1	4.0	3.1	3.1	3.4	2.7	2.5	1.7	2.8	2.4	2.4	2.9	17
Ile (20)	0.8	0.2	0.8	0.4	0.7	0.3	0.5	0.5	0.4	0.5	0.5	0.6	0.5	18
Leu (39)	14.3	13.8	14.3	10.8	13.5	11.0	12.1	13.5	11.6	12.8	12.3	12.8	12.7	10
Tyr (25)^+^	41.6	41.6	41.4	31.1	38.4	31.4	34.5	38.3	32.1	36.3	34.7	37.1	36.5	2
Phe (25)^+^	10.5	9.0	9.8	7.2	9.1	6.9	8.2	9.1	7.7	8.7	8.2	9.1	8.6	14
Trp	34.8	34.0	33.9	25.3	31.3	25.9	28.1	30.8	26.2	29.5	28.3	29.2	29.8	3
Lys (30)	13.9	13.4	13.5	9.9	12.2	9.8	11.1	12.4	10.3	11.9	11.2	11.3	11.7	12
His (10)	3.4	7.6	7.4	5.6	7.1	5.3	6.7	7.9	6.8	7.4	6.9	7.5	6.6	15
Arg	21.6	19.8	20.6	13.0	19.2	15.6	18.6	20.9	7.9	19.7	9.3	19.3	17.1	6

Rank, highest to lowest

*ND* not detected

*Combined recommendation

^+^Combined recommendation (25 total)

## Discussion

In this study, we obtained cyanobacterial material from Lake Chad that is used as a human food source and is sold through local markets. Using a variety of analytical techniques, we assessed this material for known cyanotoxins and nutritional (amino acid) content, finding it to have generally low cyanotoxin content and to be rich in amino acids.

Cyanobacterial “health food” supplements have increased in popularity (Bishop and Zubeck 2012), and with the potential to contain harmful toxins, *Spirulina* (including *Arthrospira*) and *Aphanizomenon* have been extensively studied (e.g., Roy-LaChapelle et al. 2017). Examination of such foodstuffs has identified the presence of microcystins, anatoxin-a and BMAA as examples (Rawn et al. 2007; Rellan et al. 2009; Glover et al. 2015; Roy-LaChappelle et al. 2017). Although anatoxin-a and derivatives were not found when assessing cyanobacterial supplements in the study of Rawn et al. (2007), a study by Rellan et al. (2009) confirmed their presence in 7.7% of supplements. A further study of 18 cyanobacterial supplements determined that 8 contained some amount of a range of cyanobacterial toxins (Roy-LaChapelle et al. 2017), including 5 that contained anatoxin-a and/or derivatives of this neurotoxic alkaloid. Furthermore, their assessment of microcystins in cyanobacterial supplements showed 7 of these *Spirulina* or *Aphanizomenon* supplements contained microcystins, with up to 4 individual microcystin variants found (Roy-LaChapelle et al., 2017). Of the strains tested, BMAA was found in two strains of *Aphanizomenon* and not in *Spirulina* (Roy-LaChapelle et al. 2017). Conversely, in the study of Glover et al. (2015), 5 bulk powders comprised of *Spirulina* were assessed for BMAA and isomers. The isomers *N-*(2-aminoethyl)glycine (AEG) and 2,4-diaminobutyric acid (DAB) were identified in all samples tested, and BMAA was also identified in 4 out of 5 samples at up to 0.74 μg/g (Glover et al. 2015).

We did not detect BMAA in *Arthrospira fusiformis* processed as *Dihé* wafers from Lake Chad. Although a variety of analytical methods are available for the detection of BMAA, only one has been approved by the AOAC and demonstrated to be a robust method among laboratories (Glover et al. 2015; Banack 2020). The stability and validity of using methods which do not derivatise BMAA (e.g. HILIC) have also been brought into question (Tymm et al. 2020). Therefore, although we did not detect BMAA in *Arthrospira* from Chad, this is consistent with the findings of Roy-LaChappelle et al. (2017) and used the same analytical method as that employed by the study of Glover et al. (2015).

We did find chromatographic peaks consistent with microcystins in the *Dihé* wafers, but did not detect microcystin synthetase genes using PCR in this material, although the indigenous processing of the material may have degraded the DNA, which is considered to be less stable than microcystins in dried cyanobacteria (Metcalf et al. 2012). We found four samples with trace amounts of anatoxin-a(*S*), which is a potent neurotoxin, and five samples with microcystin concentrations up to 20 μg/g, exceeding that which is considered safe for human consumption in supplements (< 1 μg/g, Marsan et al. 2017). The potential adverse health effects of DAB in the human diet have yet to be fully elucidated, but this diamino acid has been demonstrated in cyanobacterial foodstuffs (e.g., Glover et al. 2015). BMAA is also a potential cause for concern as chronic exposure to this neurotoxin is thought to be a trigger for neurodegenerative disease (Cox et al. 2016; Davis et al. 2020). The unique character of Lake Chad may account for the lack of BMAA detection reported here or may be the result of the low number of samples tested and the potential variability in concentration, as nitrogen can affect BMAA production in *Synechocystis* (Downing et al. 2011). Therefore, lakes with high nitrogen content may support cyanobacteria with BMAA at concentrations, which are low or undetectable. However, in the event of seasonal drying (Lemoalle et al. 2012), changing agricultural practices, or climate change (Coe and Foley 2001), there may still be the potential for endemic cyanobacteria to produce BMAA and future monitoring should be carried out on Lake Chad to ensure that food products from these waters are suitable for human consumption. In addition to BMAA and isomers, microcystins, and anatoxin-a(S), further research should examine the potential production of anatoxin-a by this genus as previous research has shown that microcystins and anatoxin-a are present in *Arthrospira* from African Rift Valley lakes, such as Lakes Nakuru and Bogoria, associated with periodic mass mortalities of Lesser Flamingo (Krienitz et al. 2003).

Amino acid analysis of the Lake Chad material indicates that this cyanobacterium is a rich source of amino acids and may help supplement the local population who are generally undernourished and poor (World Food Programme 2019). The absence of glutamine and asparagine in the analysis of this cyanobacterial material may have been due to degradation during acid hydrolysis. Although there is the potential for the production of toxins in Lake Chad cyanobacteria, *Spirulina* and *Arthrospira* have been shown to be good sources of protein (Becker 2007) and lipids (Bishop and Zubeck 2012). Furthermore, *Spirulina* may also be a good source of vitamins (B, E) and may help in treating diseases such as high blood pressure (Iwata et al. 1990) and diabetes (Takai et al. 1991) and as part of a balanced diet could provide essential amino acids and proteins. Assessment of commercially available *Spirulina platensis* samples showed that they could contribute significantly to amino acid RDI values from 12.8% (Lys) to 38.8% (Thr) and provide a significant input (74%) of sulfur amino acids (Misurcová et al. 2014). It would also be useful to understand why some indigenous peoples living adjacent to Lake Chad do not consume the cyanobacterial material, and if there is any seasonality to the collection and processing of the cyanobacterial blooms that are then sold to those indigenous people who do consume the material. Such seasonality could determine whether there is the potential for accumulation of cyanobacterial toxins and provide guidance for collection, processing and consumption. Observations concerning a lack of consumption from different investigators (e.g. Léonard and Compère 1967; Delpeuch et al. 1975) raise an important question: why is there no commerce among human populations immediately adjacent to Lake Chad in *Arthrospira fusiformis* or the dried *Dihé* cakes formed from it, whereas it is found commercially in markets elsewhere in Chad? It is possible that there may be cultural prescriptions against consumption of *Dihé* by those who live next to the source, possibly due to the potential presence of toxins. Only careful ethnographic studies can resolve this question, which although interesting, is beyond the scope of this study.

Studies examining the nutrition of indigenous people and their health would be useful in understanding whether cyanobacterial supplementation to the diet may be beneficial. Due to the geopolitical nature of Chad, consumption of cyanobacteria, if carefully monitored for the absence of toxins, may act as a significant health benefit and economic crop for the local population.
